# Perceptions of Patients and Physicians on Teleconsultation at Home for Diabetes Mellitus: Survey Study

**DOI:** 10.2196/27873

**Published:** 2021-11-23

**Authors:** Nazaré Rego, Helena Silva Pereira, José Crispim

**Affiliations:** 1 Escola de Economia e Gestão Universidade do Minho Braga Portugal; 2 Institute for Systems and Computer Engineering, Technology, and Science (INESC TEC) Porto Portugal; 3 Núcleo de Investigação em Políticas Económicas e Empresariais (NIPE) Escola de Economia e Gestão Universidade do Minho Braga Portugal

**Keywords:** teleconsultation, diabetes mellitus, telemedicine, eHealth, mobile phone

## Abstract

**Background:**

Diabetes mellitus (DM) is one of the most challenging diseases in the 21st century and is the sixth leading cause of death. Telemedicine has increasingly been implemented in the care of patients with DM. Although teleconsultations at home have shown to be more effective for inducing HbA_1c_ reduction than other telemedicine options, before the 2019 coronavirus disease crisis, their use had been lagging behind. Studies on physicians’ or patients’ perceptions about telemedicine have been performed independently of each other, and very few have focused on teleconsultations. In a time of great pressure for health systems and when an important portion of health care has to be assured at a distance, obtaining insights about teleconsultations at home from the stakeholders directly involved in the health care interaction is particularly important.

**Objective:**

The perceptions of patients and physicians about their intentions to use home synchronous teleconsultations for DM care are examined to identify drivers and barriers inherent to programs that involve home teleconsultations.

**Methods:**

Two identical questionnaires integrating the technology acceptance model and the unified theory of acceptance and use of technology and assessing the confidence in information and communication technology use of patients and physicians were developed. Responses by patients (n=75) and physicians (n=68) were analyzed using canonical correlation analysis.

**Results:**

Associations between predictor constructs (performance, effort, social influence, facilitating conditions, and attitude) and intention to use yielded significant functions, with a canonical *R*^2^ of 0.95 (for physicians) and 0.98 (patients). The main identified barriers to patient intention to use were the expected effort to explain the medical problem, and privacy and confidentiality issues. The major drivers were the facilitation of contact with the physician, which is beneficial to patient disease management and treatment, time savings, and reciprocity concerning physicians’ willingness to perform teleconsultations. Responses from physicians revealed an association between intention to use and the expected performance of home teleconsultations. The major barrier to intention to use expressed in physicians’ answers was doubts concerning the quality of patient examination. The major drivers were time savings, productivity increases, improvements in patient’s health and patient management, National Health System costs reduction, and reciprocity relative to patients’ willingness to engage in teleconsultations.

**Conclusions:**

To promote the use of home teleconsultations for DM, decision makers should improve patients’ health literacy so the physician–patient communication is more effective; explore information and communication technology developments to reduce current limitations of non–face-to-face examinations; ensure patient privacy and data confidentiality; and demonstrate the capabilities of home teleconsultations to physicians.

## Introduction

### Background

Diabetes mellitus (DM) is one of the most challenging diseases in the 21st century. It is the sixth leading cause of death globally [1] and continues to increase in prevalence, with macro-and microvascular complications resulting in increased disability and huge health care costs [2].

In Portugal, there were 591-699 new cases of diabetes per 100,000 inhabitants in 2015, representing an expense of 0.7%-0.9% of the Portuguese gross domestic product, and 8%-10% of the total spending on health [3]. The country has one of the highest age-adjusted prevalence of diagnosed type 1 or 2 diabetes in the population aged 20 to 79 years in Europe (9.8%) [4].

Telemedicine includes remote patient monitoring using devices (eg, mobile apps) to remotely collect and send data to health care providers, asynchronous interactions to transmit diagnostic images, vital signs, or video clips, along with patient data for later review, and synchronous live videoconferencing consultation among patients and physicians (eg, teleconsultations) or among physicians and specialist health services [5]. Information and communication technology (ICT) has been increasingly implemented in the care of people with DM to improve patient outcomes in areas such as blood glucose management, diet, medication, and exercise monitoring [6]. Although (remote) teleconsultations at home (TH) have been found to be more effective in inducing HbA_1c_ reduction when compared with other telemedicine services, such as remote telemonitoring, tele-education, and telecase management, they have been much less adopted than other forms of telemedicine in type 2 DM care [7]. Real-world data show that, before the COVID-19, teleconsultation appointments as a proportion of clinical activity ranged from 2% among a diabetic cohort to 22% among postoperative patients with hepatobiliary cancer [8,9]. In Spain, teleconsultations were used by only 7 (6.9%) of the 102 that used telemedicine in a sample of 1063 patients with type 2 DM, but obtained the highest rate of satisfaction [10]. Studies on teleconsultation in DM are scarce (eg, [11-14]). Given the use level and care potential of synchronous TH, this study investigated the necessary conditions to encourage their use.

Studies on physicians’ (eg, [15]) or patients’ perceptions (eg, [6,16]) about telemedicine have been performed independently of each other. As both groups are essential to the use of these services, this study surveyed the perceptions of the two using an identical data collection instrument and compared the results of the analysis of their responses. Identical questionnaires for both patients and physicians were used because, according to the literature [17], the factors affecting their willingness to adopt teleconsultations were the same. A canonical correlation analysis (CCA) was performed to find associations among a set of predictor constructs derived from the technology adoption literature and the *intention of use* (*IoU*) of TH by patients with DM and their physicians. CCA is a multivariate statistical technique used to study the interrelationships among sets of multiple dependent and independent variables [18]. It is an appropriate and powerful multivariate technique to identify the underlying independent relationship between the 2 sets of variables of the studied model because of the high number of variables in each construct.

### Objective

In summary, this study assesses the perspective of patients with DM and physicians regarding the drivers and barriers inherent to programs that involve patients with DM teleconsulting with their physicians from their homes.

## Methods

### Research Model

#### Overview

The questionnaires for both populations were based on the integration of the technology acceptance model (TAM) [19,20] and the unified theory of technology of acceptance and use (UTAUT) [21]. The full version of the data collection instruments (in Portuguese) can be seen in Multimedia Appendix 1 [15,17,21-25]; the English translation of the questions can be implied from the row titles of the table in Multimedia Appendix 2 [19,20,26-31].

According to Davis [19], an individual tends to use (or not use) a new technology if they identify an improvement in their professional performance. That is, if he or she easily identifies the *perceived utility*. However, the author states that the usefulness of that technology will only be recognized if the effort to learn how to use it is not very high, that is, if the use of the technology compensates for the learning effort—*perceived ease of use*. The TAM uses these 2 main constructs to influence the actual use of technology. Both have an independent effect on *IoU*, as people form intentions to adopt certain behaviors that can improve their performance at work if the effort required to learn a new technology is not considerable [19-21].

Yarbrough and Smith [32] and Holden and Karsh [26] reviewed articles on the applicability of TAM in health, reaching similar conclusions; the constructs have been repeatedly validated and the variance of the dependent variable *IoU* or *actual use of technology* has been widely explained (between 40% and 70%, depending on the study).

**Figure 1:**
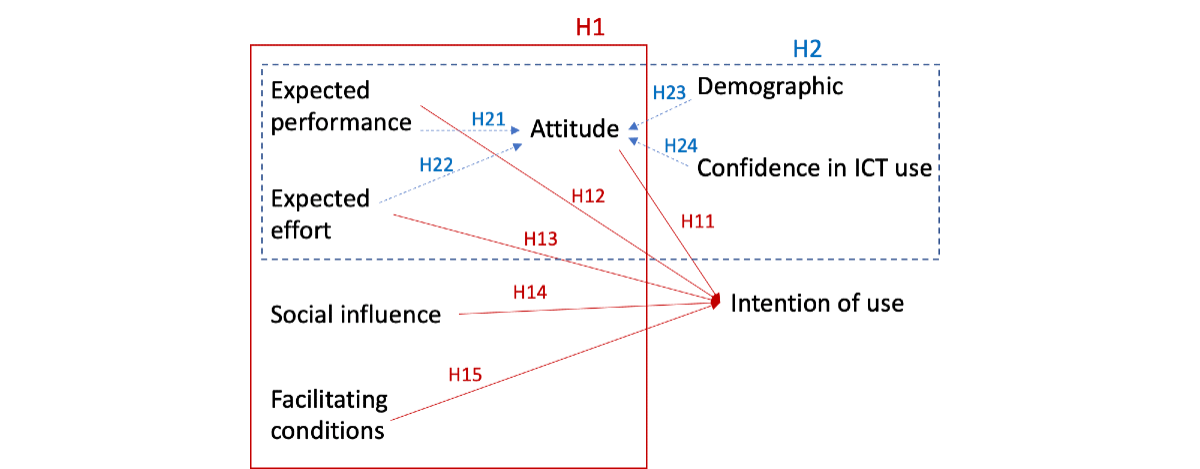
Research model. ICT: information and communication technology.

However, TAM is not very sensitive in identifying barriers to the acceptance of technology, which may influence all TAM variables. Thus, new theories explaining the acceptance of technology have emerged. One of these theories is the UTAUT, developed by Venkatesh et al [21]. UTAUT integrates the essential constructs of 8 models of technology and considers 4 constructs that directly influence the intention to use the technology: *expected performance* (P), *expected effort* (E), *social influence* (S), and *facilitating conditions* (F). The research model of this study (Figure 1) integrates TAM with UTAUT, adding an *attitude* construct [15,17,33]. In addition, we tested whether *confidence in ICT use* [27] and *demographic characteristics* were associated with *attitude*. An eventual relationship between gender and *attitude* was explored, and it was hypothesized that a younger age and higher qualifications could favor *attitude* [34].

#### Hypotheses

- H1: Do the predictors derived from the literature positively influence the IoU of TH? (suphypotheses in Table 1).
- H2: Do the predictors derived from the literature positively influence attitude (suphypotheses in Table 2).

The analysis of associations among constructs resulted in the identification of major drivers and barriers to DM (synchronous) TH.

**Table 1:** Subhypotheses of hypothesis 1.

Subhypotheses	Predictor	Effect
H11	Attitude	Positively influences the *intention of use* of teleconsultations at home
H12	Expected performance	Positively influences the *intention of use* of teleconsultations at home
H13	Expected effort	Positively influences the *intention of use* of teleconsultations at home
H14	Social influence	Positively influences the *intention of use* of teleconsultations at home
H15	Facilitating conditions	Positively influences the *intention of use* of teleconsultations at home

**Table 2:** Subhypotheses of hypothesis 2.

Subhypotheses	Predictor	Effect
H21	Expected performance	Positively influences *attitude*
H22	Expected effort (ie, perceived ease of use [20])	Positively influences *attitude*
H23	Demographic characteristics	Positively influences *attitude*
H24	Confidence in information and communication technology use	Positively influences *attitude*

### Sample and Scales

Data were collected from patients with type 1 or 2 diabetes or their caregivers, in case of child patients (75 valid responses) and physicians (68 valid responses) selected by rational choice and snowball sampling (as highly specific populations were at stake) from the north of Portugal during the fourth quarter of 2018. Concerning the patients, 51 questionnaire answers (51/75, 68% of total valid answers) were collected in-person and in paper at primary care centers belonging to the Group of Primary Care Centres of Braga, an organization that coordinates 22 primary care centers; the other were collected on web through DM patients’ associations. For physicians, the answers were collected on web with the collaboration of the same group of primary care centers. This organization sent an email with a link to the questionnaire to their physicians.

The perceptions of both groups were measured using 2 identical questionnaires based on a 7-point concordance Likert scale and a 5-point confidence Likert scale (Multimedia Appendix 2).

**Table 3:** Characteristics of the samples.

Characteristics		Patients (or caregivers; n=75) n (%)	Physicians (n=68), n (%)
**Type of respondent**			N/A^a^
	Patient	61 (81)	
	Caregiver	14 (19)	
**Gender**			
	Female	38 (51)	47 (69)
	Male	37 (49)	21 (31)
**Education**			N/A
	Basic or less	33 (44)	
	Secondary	16 (21)	
	Bachelor	8 (21)	
	Master	7 (9)	
	Opted to not respond	11 (15)	
**Medical specialty**		N/A	
	General practitioner		52 (77)
	Other		16 (23)
**Financial situation (ability to live with monthly budget)**			N/A
	Faces difficulties	8 (11)	
	Needs to manage carefully	25 (33)	
	Can go through	25 (33)	
	Goes through easily	14 (19)	
	Goes through very easily	3 (4)	
**DM^b^ type**			N/A
	1	29 (39)	
	2	44 (59)	
	Other	2 (3)	
**Treatments or disease control**			N/A
	Oral antihyperglycemic	42 (56)	
	Insulin	36 (48)	
	Antihypertensive	35 (73)	
	Antidyslipidemia	31 (41)	
	Physical exercise	41 (55)	
	Diet	44 (59)	
	Daily auto monitoring of the disease	35 (73)	
**Local for DM consultations**			
	Primary care center (public)	52 (69)	52 (77)
	Public hospital	34 (45)	21 (31)
	Private hospital	8 (11)	8 (12)
	Other (private)	3 (4)	3 (4)
**Mode of transport to consultations**			N/A
	By car	50 (67)	
	By bus	21 (28)	
	On foot	19 (26)	
	Other	3 (4)	
**Electronic devices use**			
	Computer	28 (37)	60 (88)
	Laptop	35 (47)	30 (44)
	Tablet	17 (23)	15 (22)
	Smartphone	67 (90)	49 (72)
	None	6 (8)	0 (0)
**Use of app for real-time video call**			
	Never used	28 (37)	6 (9)
	Rarely	15 (20)	26 (38)
	Once per month	7 (9)	12 (18)
	Once per week	7 (9)	5 (7)
	Several times in a week	7 (9)	9 (13)
	Everyday	11 (15)	10 (15)

^a^N/A: not applicable.

^b^DM: diabetes mellitus.

### Data Analysis

CCA was used to analyze the correlation between the set of dependent variables (*IoU* construct) and the set of predictor constructs. This method is useful when variables have multiple causes and effects, similar to the complex reality of human behavior and cognition. The computations were performed using SPSS (version 24.0; IBM Corp).

The average of the observed values is often used to form the constructs with consequent smoothing of the responses, which can lead to constructs that do not contain the variability expressed in the measurement indicators. CCA examines the relationship between the 2 observed variable sets without having this disadvantage.

Variables with a canonical correlation of 0.45 or above were considered in the final CCA model. The reliability statistics measured by Cronbach α for each construct scale were very good for *expected performance* (.87 for physicians and .83 for patients), *facilitating conditions* (.77 for physicians and .74 for patients), *attitude* (.83 for physicians and .82 for patients), and *IoU* (.91 for physicians and .78 for patients), and acceptable for *expected effort* (.60 for physicians and .56 for patients), and *social influence* (.62 for physicians and .56 for patients).

## Results

### Sample Characteristics

Survey responses of 75 patients (Table 3 and Multimedia Appendix 2) aged 10-86 years (mean 51, SD 17.1) were obtained. Of the 75 respondents, 6 (8%) had never used computers, smartphones, or tablets (Table 3). The average age of these 6 patients was 73 (SD 8.9, range 61-84) years. On average, patients had 3.1 DM consultations per year (range: 1-12). According to the respondents, DM consultations took 133 minutes on average (including travel and waiting). Of the 75 respondents, 33 (44%) patients or caregivers felt very or extremely confident and 12 (16%), moderately confident using computers or the internet. Moreover, 40% (30/75) were very or extremely confident and 24% (18/75) were moderately confident in the use of real-time video call apps. More than half had heard about telemedicine (43/75, 57% of patients) and 30% (23/75) about teleconsultations, but only 2 had participated in one in real time, and 37% (28/75) had never used an app to make a real-time video call.

In total, 68 valid responses from physicians aged 25-63 years (47/68, 69% of physicians in the interval 26-35 years) were received. Of the 68 respondents, 46 (67%) performed between 10 and 40 consultations per month, with an average duration of 23 minutes. Only 6 (9%) out of 68 physicians had never used a video call app. Moreover, 81% (55/68) felt very or extremely confident and 19% (13/68) moderately confident using computers or the internet, and 54% (37/68) were very or extremely confident. Furthermore, 28% (19/68) were moderately confident in the use of real-time video call apps. Of the 68 respondents, 33 (48.5%) physicians had never heard of TH, and 8 (12%) had already carried out synchronous teleconsultations. Although 56% (38/68) of physicians stated that they intended to use TH in follow-up consultations, 34% (25/68) answered that they would not use TH because they did not consider them a good method for health provision.

The distribution of concordance scores showed a significant variability in both groups. Wilcoxon Mann-Whitney tests identified differences between physicians and patients’ responses. Physicians had higher confidence in ICT use, but they also had higher scores for item E_3_—*Will only use TH if easy to learn* and S_1_—*if there was technical assistance*. Patients had (1) in general, a more favorable *attitude* toward TH, and higher scores in the perception that (2) *TH can invade their privacy* (*F*_3_), but (3) *be faster* (P_1_), (4) *the medical problem can be correctly understood* (E_1_) in a TH, and (5) *will have TH whenever the counterpart wants to* (S_3_).

**Figure 2:**
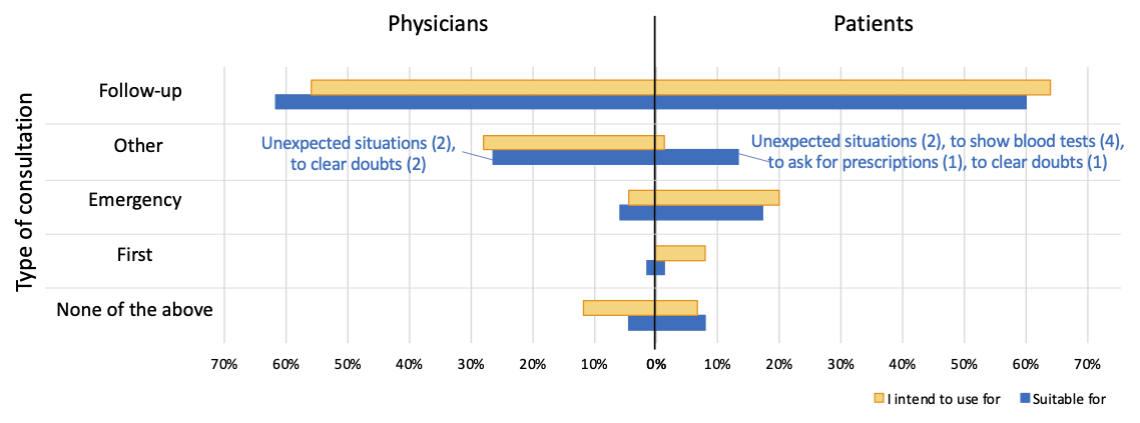
Intention of use or suitability by type of consultation.

Both patients and physicians considered follow-up to be the best purpose for TH (Figure 2).

### Hypotheses Testing

#### H1: Predictors Positively Influence the IoU of TH

##### Overview

For both physicians and patients, at least one variable of the predictors is associated with *IoU* variables (H11, H12, H13, H14, and H15 cannot be rejected).

**Figure 3:**
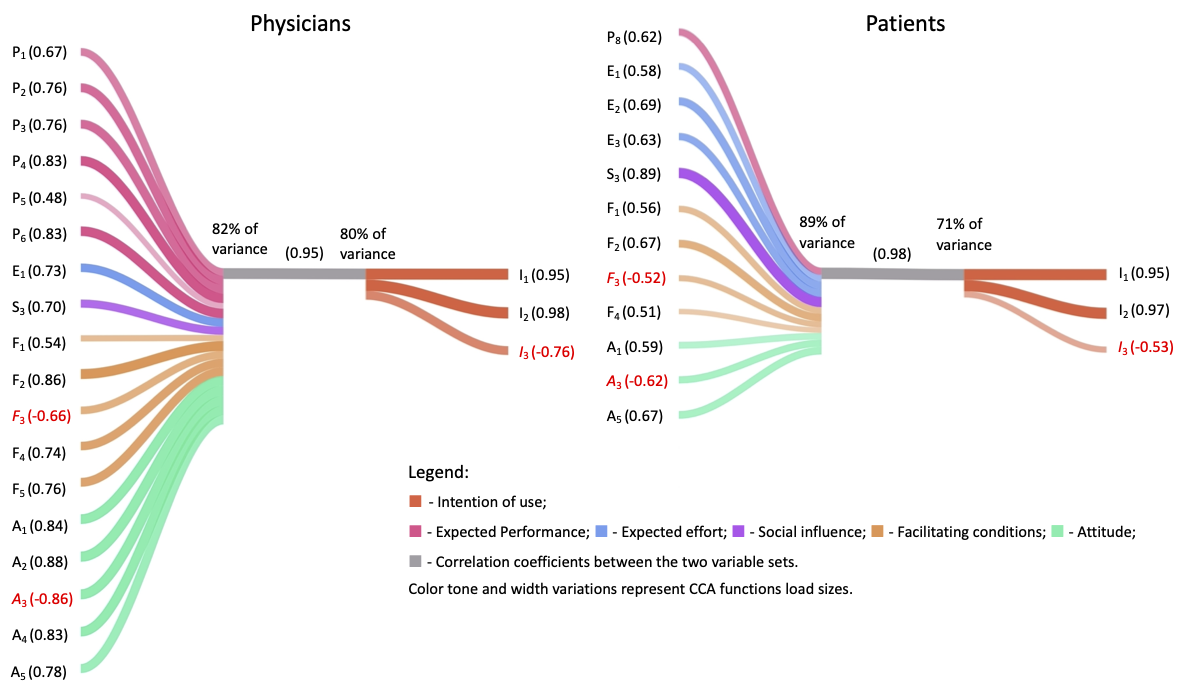
Canonical associations between predictors and intention of use. CCA: canonical correlation analysis.

Figure 3 shows the association between the latent variable *IoU* and the related covariate set of variables (predictors). Tables 4 and 5 present a validation, through comparison with the literature, of the revealed associations.

**Table 4:** Validation of the revealed associations for patients.

Canonical correlation analysis association			Variable		Literature
**Primary contributors**					
	Expected effort—IoU^a^	E_2_ (I can explain my medical problems using the computer)		Several studies found that the medium allowed patients to *open up* more than face-to-face consultations and that they felt empowered to ask more questions [35,36]	
	Social influence—IoU	S_3_ (I’ll do teleconsultations whenever the physician wants to)		To boost use, physician support and recommendation is necessary [10]	
	Facilitating conditions—IoU	F_2_ (beneficial in my management of my disease)		In Spain, most patients with type 2 diabetes (73.6%) considered that the use of telemedicine had optimized (quite a bit or a lot) the management of their disease [10]	
	Attitude—IoU	A_7_ (will not increase the provision of health care services)		Several studies found that patients were satisfied with teleconsultations, but also that they would still want the option to attend in person as they believe it to be the *gold standard* [36,37]	
**Secondary contributors**					
	Expected performance—IoU	P_8_ (allows me to save my time)		Waiting times were shorter for patients seen by teleconsultation than in face-to-face consultation as they bypassed the normal admission processes [38]	
	Effort expectancy—IoU				
		E_1_ (physician can correctly understand my medical problem)		Physical examination has become a ritual, expected, and performed as tradition rather than clinical usefulness [39]. For the time being, teleconsultations in outpatient settings are most likely to be confine to dialogue-based consultations where the need for rigorous physical examination is absent [8]	
		E_3_ (perceived as being easy to learn)		The patients were very satisfied with the technology, no major problems with its use; nearly 100% of patients reported that they would use it again and recommend it [40]	
	Facilitating conditions—IoU				
		F_1_ (can facilitate contact with the physician)		Several studies found that improved access to care was associated with patient satisfaction [40]	
		*F*_3_ *(can invade patient’s privacy)* ^b^		In a study of a teenaged population, parents are worried that the connection might not be secure enough to ensure privacy and patients fear that they might be overheard by family [36]	
		F_4_ (will not interfere with confidentiality of my health data)		The need to ensure the security and confidentiality of patient records diminishes the preference for and use of telemedicine technology [41]	
	Attitude—IoU				
		A_1_ (it is a good way to provide health care services)		In the United Kingdom, teleconsultations for acute stroke management had item values (like morbidity, mortality, and discharge rates) comparable with national standards [42,43]	
		*A*_3_ *(it is unpleasant to use teleconsultations at home)*		Several studies found that patients were satisfied with teleconsultations but also that they would still want the option to attend in person as they believe it to be the *gold standard* [36,37]	
		A_5_ (teleconsultations at home can be a supplemental health care service)		Several studies found that patients were satisfied with teleconsultations but also that they would still want the option to attend in person as they believe it to be the *gold standard* [36,37]	

^a^IoU: intention of use.

^b^Variables in *italic* had a negative sign in the predictors set.

**Table 5:** Validation of the revealed associations for physicians.

Canonical correlation analysis association		Variable	Literature
**Primary contributors**			
	Expected performance—IoU^a^		
		P_2_ (improve my productivity)	Benger et al [38] refer that teleconsultations are as much as 4 times as long as their face-to-face equivalent; however more recent studies, found them to be shorter in length (eg, [9])
		P_3_ (improves management of patient care)	Workload can be classified as the biggest workflow-related concern, as it was overrepresented in the results, being addressed in 12 of the 23 studies analyzed in the systematic literature review by Granja et al [44]
		P_4_ (improves the patient’s health)	Telehealth is a safe option for delivery of self-management support [45]
		P_6_ (improve the effectiveness of my work)	Several examples of real-world evaluations of working teleconsultation services have demonstrated that they can achieve meaningful reductions in did not attend (DNA) rates [46]
	Social influence—IoU	S_3_ (I’ll do teleconsultations whenever the patient wants to)	The literature emphasizes the role of physicians in promoting telemedicine use [10]
	Facilitating conditions—IoU		
		F_2_ (beneficial in my patient management and treatment)	DNA rates were lower (13% vs 28%) and HbA_1c_ control improved in patients that chose to attend by teleconsultation [47]
		F_4_ (will not interfere with confidentiality of the patient’s health data)	Lack of policies that guarantee the patient’s privacy and confidentiality when using and transferring information, lack of authentication by health professionals, and lack of attribution of responsibility for the quality of services are barriers to the adoption of telemedicine in health services [48]
		F_5_ (may reduce the costs of the National Health System)	In the past, the use of telemedicine was strongly dependent of technology costs (eg, [49]). Nowadays, technology allows cost savings: a report on telehealth services in Scotland found that teleconsultations for a 10-week rehabilitation course could be delivered for 3% to 10% of the cost associated with an outreach model (in which the therapist travels) or a centralized model (in which the patient travels), with savings primarily being delivered through reduced travel costs [50]
	Attitude—IoU		
		A_1_ (good way of providing health care services)	O’Cathail et al [8] summarize contradictory studies: some show that physicians lack confidence in their teleconsultation diagnosis; others assessed the concordance of diagnosis in both an inpatient and outpatient setting in neurology and found 96%-100% of cases were accurately diagnosed and managed via teleconsultation
		A_2_ (it is a good idea to use teleconsultations at home)	Amid the COVID-19 pandemic: patients can keep in touch with their routine physicians via teleconsultations; physicians could ensure drug compliance; educate patients and their caregivers; make patients aware of the common symptoms of hypoglycemia; and help patients cope with psychological problems [51]
		*A*_3_ *(it is unpleasant to use teleconsultations at home)* ^b^	The opening phase of the consultation was found to be unfamiliar, leading to interruptions and apologies on both sides whereas a dialogue flow was established [52]
		A_4_ (teleconsultation will be a common method in the future)	*No literature exploring this specific variable association was found*
		A_5_ (teleconsultations at home can be a supplemental health care service)	In some cases, the inability to perform some aspects of physical examination is likely to restrict video outpatient teleconsultations utility for more *routine* outpatient appointments [9]
**Secondary contributors**			
	Expected performance—IoU		
		P_1_ (I will be able to complete the patient’s medical consultation more quickly)	According to Benger et al [38], teleconsultations were, on average, almost twice as long as their face-to-face equivalent. However, more recent studies found them to be shorter (eg, [9])
		P_5_ (I will be able to examine the patient as well as I would during face-to-face consultations)	O’Cathail et al [8] review shows that: (1) a lack of confidence on teleconsultation diagnosis exists among professionals, (2) studies in neurology assessed the concordance of diagnosis in both an inpatient and outpatient setting and found that 96%-100% of cases were accurately diagnosed and managed via teleconsultation
	Expected effort—IoU	E_1_ (I can understand the medical problem correctly)	O’Cathail et al [8] review shows that: (1) a lack of confidence on teleconsultation diagnosis exists among professionals, (2) studies in neurology assessed the concordance of diagnosis in both an inpatient and outpatient setting and found that 96%-100% of cases were accurately diagnosed and managed via teleconsultation
	Facilitating conditions—IoU		
		F_1_ (teleconsultations at home facilitate contact with the patient)	Morris et al [47] show that, among a diabetic cohort, teleconsultation improved the DNA rate from 28% to 13% and HbA_1c_ control
		*F*_3_ *(teleconsultations at home can invade patient’s privacy)*	Some health professionals thought teleconsultations were an invasion of patients’ personal space [36]

^a^IoU: intention of use.

^b^Variables in *italic* had a negative sign in the predictors set.

##### Patients

The analysis between the set of predictor variables and *IoU* yielded one significant function with a canonical correlation of 0.98 (*P<.001*) and a canonical *R*^2^ of 0.93. The model explains about 98% (1–Wilk λ=1–0.01789) of the variance shared among the variable sets.

E_2_ (can explain medical problems using a computer), S_3_ (will have TH whenever the counterpart wants to), F_2_ (will be beneficial to manage the disease), and A_5_ (can be a supplemental care service) were the primary contributors to the predictor synthetic variable.

P_8_ (will save time), E_1_ (medical problem can be correctly understood), E_3_ (will only be used if easy to learn), F_1_ (facilitates contact with counterpart), *F*_3_ (can invade privacy), F_4_ (will not interfere with confidentiality of health data), A_1_ (is a good way to provide health care), and *A*_3_ (will be unpleasant to use TH to receive health care) were secondary contributors. The coefficient of *I*_3_ (will not be used routinely) is negative because it was negatively related to all the predictors except *F*_3_ and *A*_3_: the perception that technology *can invade patients’ privacy* (*F*_3_) and that *to use teleconsultation will be unpleasant* (*A*_3_) were positively associated with *not using teleconsultation routinely* (*I*_3_). These results generally support the theoretically expected relationships (Table 4).

##### Physicians

The analysis between the predictors and *IoU* yielded one significant function with a canonical correlation of 0.95 (*P<.001*) and a canonical *R*^2^ of 0.91. The model explains about 99% (1–Wilk λ=1–0.01010) of the variance shared between the variable sets.

The primary contributors to the predictor synthetic variable were TH can improve *my productivity* (P_2_), *management of patient care* (P_3_), *the patient’s health* (P_4_), *the effectiveness of my work* (P_6_), E_1_ (medical problem can be correctly understood), S_3_ (will have TH whenever the counterpart wants to), F_2_ (will be beneficial to manage patients and their treatment), F_4_ (will not interfere with confidentiality of health data), F_5_ (can decrease the National Health System costs), A_1_ (is a good way to provide health care services), A_2_ (is a good idea to use TH), *A*_3_ (will be unpleasant to use TH to provide health care), A_4_ (a common method for providing health care in the future), and A_5_ (can be a supplemental health care service). P_1_ (medical consultation can be completed faster), P_5_ (patient examination is as good as in face-to-face consultations), F_1_ (facilitates contact with counterpart), and *F*_3_ (can invade patient’s privacy) were secondary contributors. The structure coefficient of *I*_3_ is negative; therefore, *F*_3_ and *A_3_* are positively associated with *I*_3_. All other significant predictors were negatively associated with the *I*_3_. The results are described in Table 5.

##### Perceptions of Patients Versus Perceptions of Physicians

In terms of *expected performance* (H12), for physicians, all the variables were statistically associated with *IoU*. For the patients, only P_8_ (economy of time) was statistically associated with *IoU*, but the loading was relatively low (0.62). On the contrary, for the patients, all *expected effort* (H13) variables were statistically associated with *IoU*, whereas for physicians, only E_1_ (being able to understand the medical problem correctly).

In terms of *social influence* (H14), the theoretical relationships from the literature were not confirmed: only S_3_ (willingness to do TH whenever the counterpart wants to) exhibited statistically significant associations with *IoU*.

Relative to *facilitating conditions* (H15) and *attitude* (H11), the results for both groups were generally supportive of the theoretically expected relationships (with higher loadings for physicians). All facilitating conditions except F_5_ (TH can decrease National Health System costs) were statistically associated with *IoU* for the 2 groups. F_5_ was statistically significant only for the physicians. In terms of *attitude*, the perception that TH was *a good way to provide health care services* (A_1_) *as supplemental care* (A_5_) was positively associated, and *unpleasant to use to provide or receive health care* (*A*_3_) was negatively associated with *IoU* for both groups. Perceiving that TH will be *a common method in the future* (A_4_) was positively associated with *IoU* only for physicians.

#### H2: The Predictors Derived From the Literature Positively Influence Attitude

*Demographic data* were not associated with *attitude* and, for physicians, confidence in ICT use. H21 and H22 cannot be rejected for both groups, and H24 (specifically, relative to C3—*confidence in making videocalls*) cannot be rejected for patients.

**Figure 4:**
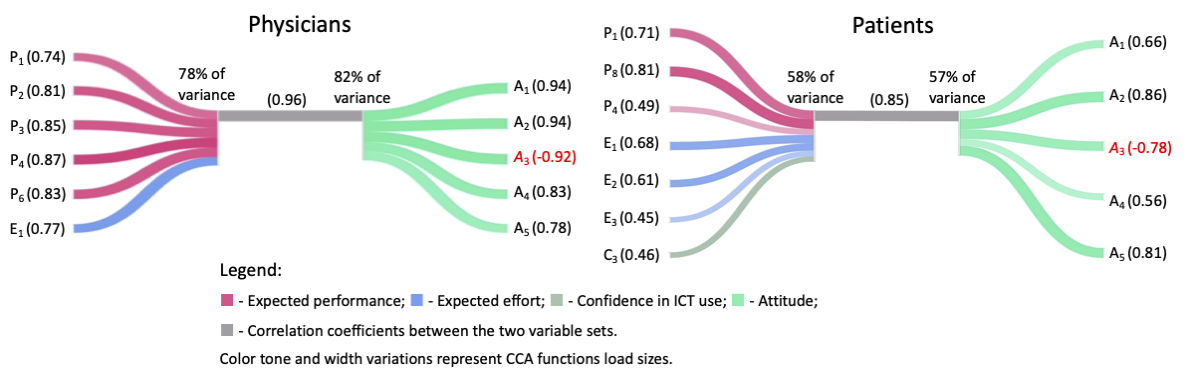
Canonical associations between predictors and attitude. CCA: canonical correlation analysis.

Figure 4 shows the canonical associations between the predictors and *attitude*. For physicians (canonical *R*^2^ of 0.96), *expected performance* and E_1_ (understanding the patient’s medical problem correctly) were associated with *attitude*, all as primary contributors. For the patients (canonical *R*^2^ of 0.85), higher associations were observed with *expected effort*, C_3_, P_1_ (medical consultation can be completed faster), P_4_ (can improve patient’s health), and P_8_ (will save patient’s time). P_1_, P_8_, and E_1_ are the primary contributors. Table 6 synthetizes the major drivers and barriers of TH for the DM.

**Table 6:** Home teleconsultation barriers and drivers.

Categories used to predict intention to use and attitude					
	Barriers			Drivers	
	Patients	Physicians	Patients		Physicians
**Expected performance**					
	None identified	P_5_—able or unable to examine the patient as well as he or she would in face-to-face consultations	P_8_—saves patient’s time		P_1_—consultation will be faster P_2_—improves physician’s productivity P_3_—improves patient management care P_4_—improves patient’s health P_6_—improves effectiveness of physician’s work
**Expected effort**					
	E_1_- physician can (not) understand patient’s medical problem correctly E_2_—patient can explain her/his medical problems using the computer E_3_—patient will only use if it is easy to learn	E_1_- physician can (not) understand patient’s medical problem correctly	None identified		None identified
**Social influence**					
	None identified	None identified	S_3_—willingness to do TH^a^ whenever the physician or patient wants to		S_3_—willingness to do TH whenever the physician or patient wants to
**Facilitating conditions**					
	*F_3_—can invade patient’s privacy^b^* F_4_—use interferes with confidentiality of patient’s health data	*F_3_—can invade patient’s privacy* F_4_—use interferes with confidentiality of patient’s health data	F_1_—facilitates contact with the patient or physician F_2_—beneficial to patient management and treatment		F_1_—facilitates contact with the patient or physician F_2_—beneficial to patient management and treatment F_5_—may reduce the costs of the National Health System
**Attitude**					
	*A_3_—it is unpleasant for physician–patient relationship* A_5_—should (only) be a supplemental health care service	*A_3_—it is unpleasant for physician–patient relationship* A_5_—should (only) be a supplemental health care service	A_1_—is a good way of providing health care services		A_1_—is a good way of providing health care services A_2_—it is a good idea to provide TH A_4_—TH will be a common method in the future
**Confidence in** **Information and communication technology** **use**					
	None identified	None identified	C_3_—confidence in making videocalls		C_3_—confidence in making videocalls

^a^TH: teleconsultations at home.

^b^Variables in italic had a negative sign in the predictors set.

## Discussion

### Principal Findings

The main contribution of this study is the identification of relationships among a set of construct predictors taken from the literature and the intention to use (synchronous) home teleconsultations. Obtaining insights about home teleconsultations from the stakeholders directly involved in the health care interaction—that is, patients and physicians, is particularly important in a time of great pressure for health systems and when an important portion of health care has to be assured at a distance.

TH appear to be safe and effective in appropriate clinical situations [9]. In addition, it should not be forgotten that more vulnerable fringes of the population would not have the resources needed for this type of consultation; for example, in our sample, some older patients had never used computers, smartphones, or tablets. Physicians and patients will likely be supportive of their use if they are offered as supplemental and in support of traditional care models rather than to replace them (most of the 2 samples agreed with this type of teleconsultation; Multimedia Appendix 2, variable A_5_). This result is in line with the findings of Gilbert et al [37], from the patient’s perspective, and Greenhalgh et al [9], from that of the physicians.

Health illiteracy and the physical examination *ritual* (referred by Haig-Ferguson et al [36] in the context of a pediatric chronic fatigue service) may explain why patients see TH only as an extra health care service. Patients should be encouraged and supported for their use.

Expected performance factors (time savings, increased productivity or efficiency, better disease management, health improvement, and quality of the clinical examination) were the most important factors for *intention to use* among physicians, which is in line with the literature (Table 5). On the contrary, except for time savings, patients’ perceptions did not reveal an association between performance variables and *intention to use*.

Another difference concerns the *expected effort* needed to use TH. For the patients, explaining *and being understood when communicating their medical problems using the computer* and *the technology being easy to learn* are positively associated with *intention to use.* This type of concern has been identified in the literature [8]. For physicians, these factors are not related to *the intention to use*, except for the necessary effort to *understand the patient’s problem*. In their systematic literature review on physicians’ eHealth adoption, Granja et al [44] concluded that the major facilitators of eHealth are the quality of the diagnosis and patient-centered care.

For both groups, the only *social influence* variable associated with *intention to use* was the willingness of patients or physicians to participate in TH if their counterpart wants to, which means that each group can encourage the use of the other. The literature has only referred to the importance of physicians’ recommendations for teleconsultation [10].

For both patients and physicians, all *facilitating conditions* and *attitude* variables toward using TH were associated with *intention to use*, which is in accordance with the literature (Tables 4 and 5).

Curiously, contrary to the evidence described in the literature (eg, [53]), this study did not find a statistically significant association between *demographic data* or *confidence in ICT use* and *attitude* for any of the groups, with the exception of confidence in using video calls for patients. The current COVID-19 pandemic has led many people to communicate through videoconferencing. Given that our results point to a positive association between confidence in the use of video calls and *attitude*, the pandemic situation may have been a booster for patients’ TH adherence. For teleconsultations to be an effective addition to health services beyond COVID-19 they should be considered not only as a technological issue, but also as a complex organizational change problem [54]; from the aspects raised by the authors, we would like to highlight the need for adjusted legal frameworks and reimbursement schemes.

Major barriers to TH use identified were: (1) the inability to correctly understand the medical problem, (2) threats to patient privacy, (3) health data confidentiality, (4) unpleasantness of TH to provide or receive health care, and (5) type of TH use (supplemental care service). On the basis of the perceptions of patients, costs do not seem to be a barrier to TH use, contrary to what has been described in the literature [8]. Probably, this result was observed because, nowadays, technological devices that can be used to make teleconsultations (smartphones, tablets, laptops, etc) are easily available in most of the situations. As digital interaction generally has insignificant costs for patients, cost is highly dependent on the existence of the technology.

The major identified TH drivers were (1) the perception that they facilitate contact, and (2) the fact that the use by each group was highly influenced by the other. Furthermore, physicians are very sensitive to issues related to the performance and quality of service.

The sampling methods limit the generalizability of the results. The composition of the patients’ sample in terms of age and education was similar to that of the general population in northern Portugal. However, the proportion of patients with type 1 (type 2) DM in the sample is higher (lower) than expected in the population [4]. Thus, the patients’ population may be, on average, older and less educated than the sample in this study. As the data concerning the perceptions of physicians were collected on web, the sample may be, on average, more technology favorable than the population. Nevertheless, both samples included individuals ranging from more positive to more skeptic about TH. In addition, the results were compared with and discussed against the findings of related studies.

A CCA revealed a strong association between the predictors and the set of dependent variables, in line with the literature. The data analysis included a joint critical comparison of the perceptions of patients and physicians. To promote the use of home teleconsultations for DM, decision makers should: (1) improve patient health literacy, as the inability to explain medical problems correctly emerges as a barrier to teleconsultation use; (2) explore ICT developments to reduce current limitations of non–face-to-face examination; (3) ensure patient privacy and data confidentiality; and (4) demonstrate the capabilities of home teleconsultations to physicians, namely, in terms of the ability to enhance patient–physician communication and to educate patients and their caregivers toward a better management of the disease.

### Conclusions

In the future, it would be interesting that research about teleconsultations acceptance incorporated sustainability related aspects like, for example, fuel consumption, carbon emissions, and loss of work productivity. A recent review [55] concluded that, as patients, health care organizations, and nations continue to look toward video consultations as an alternative, it is essential to continue to theorize in this domain.

## Appendix

Multimedia Appendix 1 Questionnaires to collect perceptions of diabetes mellitus patients and physicians about teleconsultations at home.

Multimedia Appendix 2 Constructs.

## Abbreviations

CCA: canonical correlation analysis
DM: diabetes mellitus
DNA: did not attend
ICT: information and communication technology
IoU: intention of use
TAM: technology acceptance model
TH: teleconsultations at home
UTAUT: unified theory of acceptance and use of technology
