# Supplementary material for: Perceptions of Patients and Physicians on Teleconsultation at Home for Diabetes Mellitus: Survey Study
Source: JMIR Hum Factors. 2021 Nov 23;8(4):e27873. doi: 10.2196/27873 (PMC8663635; doi:10.2196/27873)
Supplement: Multimedia Appendix 1 [file humanfactors_v8i4e27873_app1.pdf]

PATIENTS' QUESTIONNAIRE

A perspetiva dos doentes de Diabetes *Mellitus* no que respeita à realização de teleconsultas em ambiente domiciliário

Este questionário enquadra-se numa investigação no âmbito de uma tese de Mestrado em Estudos de Gestão, realizada na Escola de Economia e Gestão da Universidade do Minho. Os resultados obtidos serão utilizados apenas para fins académicos, sendo realçado que as suas respostas representam apenas a sua opinião individual.

O questionário é anónimo, não devendo por isso colocar a sua identificação em nenhuma das folhas, nem assinar o questionário.

Não existem respostas certas ou erradas. Por isso, solicitamos-lhe que responda de forma espontânea e sincera a todas as questões. Na maioria das questões terá apenas de assinalar com uma cruz a sua opção de resposta.

Obrigado pela sua colaboração.

Enquadramento do tema

A Telemedicina corresponde à oferta de serviços ligados aos cuidados com a saúde, prestados por profissionais da área da saúde, usando tecnologias de informação e de comunicação – como um computador, *tablet* e telemóvel – para troca de informações válidas para diagnóstico, prevenção e tratamento de doenças, tendo sempre como objetivo principal a melhoria da saúde do Indivíduo e da sua Comunidade (World Health Organization, 1998).

Em Portugal, já são realizadas teleconsultas em tempo real, onde o utente se encontra junto do seu médico, no Centro de Saúde ou Hospital, e comunica com outro médico especialista que se encontra a trabalhar noutro Hospital, utilizando o computador com microfone e câmara web do gabinete médico, para trocar informações audiovisuais e de dados com o outro médico, sendo obrigatório o registo no equipamento e no processo clínico do doente (Ministério da Saúde, 2013). No entanto, ainda não são realizadas teleconsultas em ambiente domiciliário, onde o doente pode comunicar com o seu médico sem se ter de deslocar ao Centro de Saúde ou Hospital, ou seja, pode realizar a consulta com o seu médico a partir de sua casa ou de outro local. Para tal, seria necessário que o doente tivesse um computador/tablet/telemóvel, com câmara web e com ligação à Internet, onde seria instalado um programa informático ou uma aplicação informática (no caso do tablet e telemóvel) que lhe permitisse ver, falar e trocar informações com o seu médico.

Como tal, esta investigação foca-se em estudar a perspetiva dos médicos e doentes de *Diabetes Mellitus* no que respeita a oportunidades e barreiras inerentes à implementação de programas que permitam ao doente realizar teleconsultas com o seu médico, a partir de sua casa, isto é, que lhe permitam realizar teleconsultas em ambiente domiciliário.

Dados Sócio-Demográficos

1. Idade: _______anos
2. Sexo: ☐Feminino ☐Masculino
3. Habilitações Literárias:

☐1º ciclo (antiga 4ª classe) ☐2ºciclo (antigo 6ºano) ☐3ºciclo (9º ano)

☐Ensino secundário (12ºano) ☐Licenciatura ☐Mestrado

☐Doutoramento

4. Concelho de Residência: ____
5. Como avalia a sua situação financeira atual?

☐Tenho dificuldades para me sustentar durante o mês
☐Tenho que gerir muito bem o meu dinheiro para me conseguir sustentar durante o mês
☐Sou capaz de me sustentar durante o mês
☐Tenho facilidade para me sustentar durante o mês
☐Tenho bastante facilidade em me sustentar durante o mês

6. É doente ou cuidador? ☐Doente ☐Cuidador

No caso de ser cuidador, por favor indique a idade do doente: _______ anos

7. Qual o tipo de diabetes *Mellitus*?

☐Diabetes Tipo 1 ☐Diabetes tipo 2

☐Diabetes Gestacional ☐Outros tipos específicos de diabetes

8. Que tipo de tratamento efetua? *(pode selecionar mais que uma opção)*

☐Insulina ☐Antidiabéticos orais ☐Antidislipidémicos (colesterol elevado)

☐Antihipertensivos (pressão arterial elevada) ☐Alimentação controlada ☐Exercício físico

☐Autovigilância diária (medição da glicémia) ☐Anto-controlo (ajuste da dose de insulina, da alimentação e/ou da atividade física)

9. Quantas consultas para controlo de diabetes, programadas ou não programadas, tem por ano? (aproximadamente) _________________________

10. Concelho onde tem consultas: *(selecione o local onde tem consultas, indicando o concelho respetivo. Pode selecionar mais que uma opção)*

☐Centro de Saúde: ____
☐Hospital: ____

11. Qual o meio de transporte que utiliza para se deslocar para as consultas? *(Pode selecionar mais que uma opção)*

☐Carro ☐Autocarro ☐Táxi

☐Ambulância para transporte de doentes ☐A pé ☐Mota

☐Outro: ____

12. Quanto tempo despende, aproximadamente, numa consulta? *(incluindo tempo de deslocação, espera e consulta)* ______________________________

13. Que dispositivos informáticos e de comunicação utiliza? *(Pode selecionar mais que uma opção)*

☐Computador fixo ☐Computador Portátil ☐Tablet

☐Telemóvel (Smartphone) ☐Não utilizo nenhum

14. Em que dispositivos informáticos e de comunicação utiliza Internet? *(Pode selecionar mais que uma opção)*

☐Computador fixo ☐Computador Portátil ☐Tablet

☐Telemóvel (Smartphone) ☐Não uso Internet

15. Numa escala de 1 a 5, quão confiante se sente na utilização de um computador?

**1 – Nada confiante**	**2 – Pouco confiante**	**3 – Moderadamente confiante**	**4 – Muito confiante**	**5 – Extremamente confiante**
☐	☐	☐	☐	☐

16. Numa escala de 1 a 5, quão confiante se sente na utilização da Internet?

**1 – Nada confiante**	**2 – Pouco confiante**	**3 – Moderadamente confiante**	**4 – Muito confiante**	**5 – Extremamente confiante**
☐	☐	☐	☐	☐

17. Quantas vezes utiliza aplicações de videochamadas em tempo real? (*por exemplo: Skype*, *FaceTime*, *Messenger*, *Google Hangouts*, *etc*). *(Selecione APENAS UMA opção)*

☐Nunca utilizei
☐Muito esporadicamente
☐Uma vez por mês
☐Uma vez por semana
☐Várias vezes por semana
☐Todos os dias

18. Numa escala de 1 a 5, quão confiante se sente na utilização aplicações de videochamadas em tempo real? (*p.ex.: Skype*, *FaceTime*, *Messenger*, *Google Hangouts*, *etc*).

**1 – Nada confiante**	**2- Pouco confiante**	**3 – Moderadamente confiante**	**4 – Muito confiante**	**5 – Extremamente confiante**
☐	☐	☐	☐	☐

19. Já alguma vez ouviu falar de “Telemedicina”? ☐Sim ☐Não
20. Já alguma vez realizou uma **teleconsulta em tempo real***? ☐Sim ☐Não
    Se sim, quantas? ________________________
21. Já alguma vez ouviu falar de **teleconsulta em ambiente domiciliário****? ☐Sim ☐Não

Teleconsulta em tempo real –** *consulta fornecida por um médico distante do utente, através de um computador, com a presença do doente junto de outro médico, numa outra localização (por exemplo, Centro de Saúde ou Hospital).**

*****Teleconsulta em ambiente domiciliário –** *consulta fornecida por um médico distante do utente, através de um computador, onde o doente pode estar em sua casa ou noutro local à escolha, não necessitando de se dirigir ao Centro de Saúde ou Hospital.*

-----------------O QUESTIONÁRIO CONTINUA NA PRÓXIMA PÁGINA-----------------

Teleconsultas em Ambiente Domiciliário

Nesta secção são apresentadas várias afirmações sobre teleconsultas em ambiente domiciliário. Por favor, leia os itens e responda honesta e verdadeiramente, assinalando o quadrado da resposta que mais lhe parece adequada a cada questão, segundo a escala de 1-7, abaixo apresentada. Escolha APENAS UMA opção para cada afirmação.

1 – DISCORDO TOTALMENTE; 2 – DISCORDO BASTANTE; 3 – DISCORDO; 4 – NEM CONCORDO NEM DISCORDO; 5 – CONCORDO; 6 – CONCORDO BASTANTE; 7 – CONCORDO TOTALMENTE

	1	2	3	4	5	6	7
**18.** As teleconsultas em ambiente domiciliário podem economizar tempo aos profissionais de saúde (Demiris et al., 2000).	☐	☐	☐	☐	☐	☐	☐
**19.** As teleconsultas em ambiente domiciliário **não** me possibilitam economizar dinheiro (Demiris et al., 2000).	☐	☐	☐	☐	☐	☐	☐
**20.** As teleconsultas em ambiente domiciliário correspondem a um bom método de prestação de cuidados de saúde (Demiris et al., 2000).	☐	☐	☐	☐	☐	☐	☐
**21.** As teleconsultas em ambiente domiciliário permitem-me economizar o meu tempo (Demiris et al., 2000).	☐	☐	☐	☐	☐	☐	☐
**22.** As teleconsultas em ambiente domiciliário podem melhorar a minha saúde (Demiris et al., 2000).	☐	☐	☐	☐	☐	☐	☐
**23.** Nas teleconsultas em ambiente domiciliário, um profissional de saúde **não** me pode examinar tão bem quanto examinaria nas consultas presenciais (Demiris et al., 2000).	☐	☐	☐	☐	☐	☐	☐
**24.** As teleconsultas em ambiente domiciliário podem facilitar o contacto com o profissional de saúde (Demiris et al., 2000).	☐	☐	☐	☐	☐	☐	☐
**25.** Apenas utilizarei teleconsultas em ambiente domiciliário se houver apoio e assistência técnica, em caso de dificuldades no uso (Kohnke et al., 2014).	☐	☐	☐	☐	☐	☐	☐
**26.** O profissional de saúde poderá entender corretamente o meu problema médico, através do computador (Demiris et al., 2000).	☐	☐	☐	☐	☐	☐	☐
**27.** Sou capaz de explicar os meus problemas médicos através do computador (Demiris et al., 2000).	☐	☐	☐	☐	☐	☐	☐

	1	2	3	4	5	6	7
**28.** Aprender a utilizar a tecnologia necessária para realizar teleconsultas em ambiente domiciliário será fácil para mim (Venkatesh et al., 2003).	☐	☐	☐	☐	☐	☐	☐
**29.** Apenas realizarei teleconsultas em ambiente domiciliário se a tecnologia for fácil de usar (Kifle et al., 2010).	☐	☐	☐	☐	☐	☐	☐
**30.** É uma boa ideia recorrer a teleconsultas em ambiente domiciliário como uma forma de receber serviços de saúde (Venkatesh et al., 2003).	☐	☐	☐	☐	☐	☐	☐
**31.** Será desagradável recorrer a teleconsultas em ambiente domiciliário para receber cuidados de saúde (Venkatesh et al., 2003).	☐	☐	☐	☐	☐	☐	☐
**32.** O uso de teleconsultas em ambiente domiciliário poderá tornar-se benéfico na gestão e tratamento da minha doença (Venkatesh et al., 2003).	☐	☐	☐	☐	☐	☐	☐
**33.** O serviço de telemedicina em ambiente domiciliário poderá violar a minha privacidade (Demiris et al., 2000).	☐	☐	☐	☐	☐	☐	☐
**34.** O uso de tecnologia nas teleconsultas em ambiente domiciliário **não** irá interferir com a confidencialidade dos meus dados de saúde (Demiris et al., 2000).	☐	☐	☐	☐	☐	☐	☐
**35.** As teleconsultas em ambiente domiciliário serão, no futuro, um método comum de prestação de serviços (Demiris et al., 2000).	☐	☐	☐	☐	☐	☐	☐
**36.** As teleconsultas em ambiente domiciliário poderão ser um serviço de prestação de cuidado de saúde extra, relativamente aos cuidados de saúde que já recebo (Demiris et al., 2000).	☐	☐	☐	☐	☐	☐	☐
**37.** As teleconsultas em ambiente domiciliário poderão reduzir os custos do Sistema Nacional de Saúde (Demiris et al., 2000).	☐	☐	☐	☐	☐	☐	☐
**38.** As teleconsultas em ambiente domiciliário **não** irão aumentar a oferta de serviços de prestação de cuidados de saúde (Demiris et al., 2000).	☐	☐	☐	☐	☐	☐	☐
**39.** Tenciono recorrer a teleconsultas em ambiente domiciliário quando esta tecnologia e serviço estiver disponível no meu centro de saúde/hospital (Venkatesh et al., 2003).	☐	☐	☐	☐	☐	☐	☐

	1	2	3	4	5	6	7
**40. Não** pretendo recorrer de modo rotineiro a teleconsultas em ambiente domiciliário para receber serviços de prestação de cuidados de saúde (Venkatesh et al., 2003).	☐	☐	☐	☐	☐	☐	☐
**41.** Tenciono recorrer a teleconsultas em ambiente domiciliário quando esta tecnologia for utilizada pelo meu médico (Venkatesh et al., 2003).	☐	☐	☐	☐	☐	☐	☐
**42.** Tenciono recorrer a teleconsultas em ambiente domiciliário para receber serviços de tratamento de saúde, sempre que o meu médico o pretenda (Venkatesh et al., 2003).	☐	☐	☐	☐	☐	☐	☐
**43. Não** pretendo recorrer a teleconsultas em ambiente domiciliário se implicarem custos adicionais, em Saúde, para mim (Rho et al., 2014).	☐	☐	☐	☐	☐	☐	☐
**27**. Tenciono recorrer a teleconsultas em ambiente domiciliário para fornecer cuidados de saúde, se as pessoas que influenciam o meu comportamento me incentivarem a tal (Venkatesh et al., 2003).	☐	☐	☐	☐	☐	☐	☐

Nesta secção serão apresentadas duas questões de escolha múltipla sobre teleconsultas em ambiente domiciliário. Por favor, leia os itens e responda honesta e verdadeiramente, assinalando o(s) botão(ões) da(s) opções que mais lhe parece(m) adequada(s) a cada questão. Pode assinalar mais que uma opção.

28. As teleconsultas em ambiente domiciliário correspondem a um bom método de prestação de cuidados de saúde ao ser utilizado em:

☐Primeira consulta

☐Consultas de acompanhamento/controlo

☐Consultas de emergência/não programadas

☐Não considero um bom método de prestação de cuidados de saúde

☐Outro.

Qual?____

29. Pretendo recorrer a teleconsultas em ambiente domiciliário em:

☐Primeira consulta

☐Consultas de acompanhamento/controlo

☐Consultas de emergência/não programadas

☐Não pretendo recorrer a teleconsultas em ambiente domiciliário

☐Outro.

Qual?____

-----------------FIM DO QUESTIONÁRIO-----------------

MUITO OBRIGADA PELA SUA COLABORAÇÃO!

Referências bibliográficas

Demiris, G., Speedie, S., & Finkelstein, S. (2000). A questionnaire for the assessment of patients' impressions of the risks and benefits of home telecare. *Journal of Telemedicine and Telecare*, *6*, 278–284. https://doi.org/10.1258/1357633001935914

Kifle, M., Payton, F. C., Mbarika, V., & Meso, P. (2010). Transfer and Adoption of Advanced Information Technology Solutions in Resource-Poor Environments: The Case of Telemedicine Systems Adoption in Ethiopia. *Telemedicine and E-Health*, *16*(3), 327–343. https://doi.org/10.1089/tmj.2009.0008

Kohnke, A., Cole, M. L., & Bush, R. (2014). Incorporating UTAUT predictors for understanding home care patients' and clinician's acceptance of healthcare telemedicine equipment. *Journal of Technology Management and Innovation*, *9*(2), 29–41. https://doi.org/10.4067/S0718-27242014000200003

Ministério da Saúde. (2013). Despacho n.o 3571/2013. Diário Da República, 2.a série(N.o 46), 8325–8326. Retrieved from https://dre.pt/dre/detalhe/despacho/3571-2013-1759945

Rho, M. J., Choi, I., & Lee, J. (2014). Predictive factors of telemedicine service acceptance and behavioral intention of physicians. International Journal of Medical Informatics, 83(8), 559–571. https://doi.org/10.1016/j.ijmedinf.2014.05.005

Venkatesh, V., Morris, M. G., Davis, G. B., & Davis, F. D. (2003). User acceptance of information technology: toward a unified view. *MIS Quarterly*, *27*(3), 425–478. https://doi.org/10.2307/30036540

World Health Organization. (1998). A Health Telematics Policy. Report of the WHO Group Consultation on Health Telematics - A Health Telematics Policy. Retrieved from https://apps.who.int/iris/handle/10665/63857

A perspetiva dos médicos no que respeita à realização de teleconsultas em ambiente domiciliário a doentes de Diabetes *Mellitus*

Este questionário enquadra-se numa investigação no âmbito de uma tese de Mestrado em Estudos de Gestão, realizada na Escola de Economia e Gestão da Universidade do Minho. Os resultados obtidos serão utilizados apenas para fins académicos, sendo realçado que as suas respostas representam apenas a sua opinião individual.

O questionário é anónimo, não devendo por isso colocar a sua identificação em nenhuma das folhas, nem assinar o questionário.

Não existem respostas certas ou erradas. Por isso, solicitamos-lhe que responda de forma espontânea e sincera a todas as questões. Na maioria das questões terá apenas de assinalar com uma cruz a sua opção de resposta.

Obrigado pela sua colaboração.

Enquadramento do tema

A Telemedicina corresponde à oferta de serviços ligados aos cuidados com a saúde, prestados por profissionais da área da saúde, usando tecnologias de informação e de comunicação para o intercâmbio de informações válidas para diagnóstico, prevenção e tratamento de doenças, tendo sempre como objetivo principal a melhoria da saúde do Indivíduo e da sua Comunidade (World Health Organization, 1998).

Em Portugal, já são realizadas teleconsultas em tempo real, onde o utente se encontra junto ao seu médico, no Centro de Saúde ou Hospital, e comunica com outro médico especialista que se encontra a trabalhar noutro Hospital, com recurso à utilização de comunicações interativas, audiovisuais e de dados, e com registo obrigatório no equipamento e no processo clínico do doente (Ministério da Saúde, 2013). No entanto, ainda não são realizadas teleconsultas em ambiente domiciliário, onde o doente pode comunicar com o seu médico sem se ter de deslocar ao Centro de Saúde ou Hospital, ou seja, pode realizar a consulta com o seu médico a partir de sua casa, utilizando um computador, com câmara web e com ligação à Internet, e equipado com um programa informático onde pode ver, falar e trocar informações com o seu médico.

Como tal, esta investigação foca-se em estudar a perspetiva dos médicos e doentes de *Diabetes Mellitus* no que respeita a oportunidades e barreiras inerentes à implementação de programas que permitam ao doente realizar teleconsultas com o seu médico, a partir de sua casa, isto é, teleconsultas em ambiente domiciliário.

Dados Sócio-Demográficos

1. Idade:________anos
2. Sexo: ☐Feminino ☐Masculino
3. Especialidade: ____

4. Concelho de Residência:
____
5. Quantos doentes de diabetes *mellitus* acompanha, por mês?

   ☐0 ☐21-30

   ☐1-5 ☐31-40

   ☐6-10 ☐41-50

   ☐11-20 ☐ >50

6. Quantas consultas para controlo de diabetes, programadas ou não programadas, efetua por mês?

   ☐0 ☐51-70

   ☐1-10 ☐70-100

   ☐11-30 ☐>100

   ☐31-50

7. Concelho onde tem consultas: *(selecione o local onde tem consultas, indicando o concelho respetivo. Pode selecionar mais que uma opção)*

☐Centro de Saúde:
☐Hospital Público:
☐Hospital Privado:
☐Consultório Privado:

8. Quanto tempo despende, aproximadamente, numa consulta com um doente diabético? min.
9. Que dispositivos informáticos e de comunicação utiliza, no dia a dia? *(Pode selecionar mais que uma opção)*

   ☐Computador fixo ☐Computador Portátil ☐Tablet

   ☐Telemóvel (Smartphone) ☐Não utilizo nenhum

10. Em que dispositivos informáticos e de comunicação utiliza Internet? *(Pode selecionar mais que uma opção)*

    ☐Computador fixo ☐Computador Portátil ☐Tablet

    ☐Telemóvel (Smartphone) ☐Não utilizo Internet

11. Numa escala de 1 a 5, quão confiante se sente na utilização de um computador?

**1 – Nada confiante**	**2 – Pouco confiante**	**3 – Moderadamente confiante**	**4 – Muito confiante**	**5 – Extremamente confiante**
☐	☐	☐	☐	☐

12. Numa escala de 1 a 5, quão confiante se sente na utilização da Internet?

1 – Nada confiante	2 – Pouco confiante	3 – Moderadamente confiante	4 – Muito confiante	5 – Extremamente confiante
☐	☐	☐	☐	☐

13. Quantas vezes utiliza aplicações de videochamadas em tempo real? (*por exemplo: Skype*, *FaceTime*, *Messenger*, *Google Hangouts*, *etc*)

☐Nunca utilizei
☐Muito esporadicamente
☐Uma vez por mês
☐Uma vez por semana
☐Várias vezes por semana
☐Todos os dias

14. Numa escala de 1 a 5, quão confiante se sente na utilização aplicações de videochamadas em tempo real? (*p.ex.: Skype*, *FaceTime*, *Messenger*, *Google Hangouts*, *etc*)

1 – Nada confiante	2- Pouco confiante	3 – Moderadamente confiante	4 – Muito confiante	5 – Extremamente confiante
☐	☐	☐	☐	☐

15. Já alguma vez ouviu falar de "Telemedicina"? ☐Sim ☐Não
16. Já alguma vez realizou uma teleconsulta em tempo real? ☐Sim ☐Não
    Se sim, quantas?
    ☐ <10 ☐71-100
    ☐10-30 ☐>100
    ☐31-50
    ☐51-70
17. Já alguma vez ouviu falar de teleconsultas em ambiente domiciliário? ☐Sim ☐Não

------------------O QUESTIONÁRIO CONTINUA NA PRÓXIMA PÁGINA-------------------

Teleconsultas em Ambiente Domiciliário

Nesta secção são apresentadas várias afirmações sobre teleconsultas em ambiente domiciliário. Por favor, leia os itens e responda honesta e verdadeiramente, assinalando o botão da resposta que mais lhe parece adequada a cada questão, segundo a escala de 1-7, abaixo apresentada. Escolha APENAS UMA opção para cada afirmação.

1 – DISCORDO TOTALMENTE; 2 – DISCORDO BASTANTE; 3 – DISCORDO; 4 – NEM CONCORDO NEM DISCORDO; 5 – CONCORDO; 6 – CONCORDO BASTANTE; 7 – CONCORDO TOTALMENTE

	1	2	3	4	5	6	7
**18.** Ao recorrer a Teleconsultas em ambiente domiciliário, poderei completar mais rapidamente o atendimento do doente (Venkatesh et al., 2003).	☐	☐	☐	☐	☐	☐	☐
**19.** Ao efetuar teleconsultas em ambiente domiciliário poderei melhorar a minha produtividade (Venkatesh et al., 2003).	☐	☐	☐	☐	☐	☐	☐
**20.** As teleconsultas em ambiente domiciliário poderão facilitar a minha gestão de tratamento do doente (Venkatesh et al., 2003).	☐	☐	☐	☐	☐	☐	☐
**21.** As teleconsultas em ambiente domiciliário correspondem a um bom método de prestação de cuidados de saúde (Demiris et al., 2000).	☐	☐	☐	☐	☐	☐	☐
**22.** As teleconsultas em ambiente domiciliário poderão melhorar a saúde do doente (Demiris et al., 2000).	☐	☐	☐	☐	☐	☐	☐
**23.** Nas teleconsultas em ambiente domiciliário, **não** poderei examinar tão bem o doente quanto examinaria nas consultas presenciais (Demiris et al., 2000).	☐	☐	☐	☐	☐	☐	☐
**24.** As teleconsultas em ambiente domiciliário poderão facilitar o contacto com o doente (Demiris et al., 2000).	☐	☐	☐	☐	☐	☐	☐
**25.** As teleconsultas em ambiente domiciliário **não** vão melhorar a eficácia do meu trabalho (Demiris et al., 2000).	☐	☐	☐	☐	☐	☐	☐
**26.** Poderei entender corretamente o problema médico do doente, através do computador (Demiris et al., 2000).	☐	☐	☐	☐	☐	☐	☐
**27.** Aprender a utilizar a tecnologia necessária para realizar teleconsultas em ambiente domiciliário será fácil para mim (Venkatesh et al., 2003).	☐	☐	☐	☐	☐	☐	☐
**28.** Apenas realizarei teleconsultas em ambiente domiciliário se a tecnologia for fácil de usar (Kifle et al., 2010).	☐	☐	☐	☐	☐	☐	☐

	1	2	3	4	5	6	7
**29.** É uma boa ideia recorrer a teleconsultas em ambiente domiciliário como uma forma de prestar serviços de cuidados de saúde ao doente (Venkatesh et al., 2003).	☐	☐	☐	☐	☐	☐	☐
**30.** Será desagradável recorrer a teleconsultas em ambiente domiciliário durante a prestação de serviços de saúde ao doente (Venkatesh et al., 2003).	☐	☐	☐	☐	☐	☐	☐
**31.** O uso de teleconsultas em ambiente domiciliário poderá tornar-se benéfico na minha gestão de doentes e tratamento destes (Venkatesh et al., 2003).	☐	☐	☐	☐	☐	☐	☐
**32.** O serviço de teleconsultas em ambiente domiciliário poderá violar a privacidade do doente (Demiris et al., 2000).	☐	☐	☐	☐	☐	☐	☐
**33.** O uso de tecnologia nas teleconsultas em ambiente domiciliário **não** irá interferir com a confidencialidade dos dados de saúde do doente (Demiris et al., 2000).	☐	☐	☐	☐	☐	☐	☐
**34.** As teleconsultas em ambiente domiciliário serão, no futuro, um método comum de prestação de serviços (Demiris et al., 2000).	☐	☐	☐	☐	☐	☐	☐
**35.** As teleconsultas em ambiente domiciliário poderão ser um serviço de prestação de cuidado de saúde extra, relativamente aos cuidados de saúde que já forneço (Demiris et al., 2000).	☐	☐	☐	☐	☐	☐	☐
**36.** As teleconsultas em ambiente domiciliário poderão reduzir os custos do Sistema Nacional de Saúde (Demiris et al., 2000).	☐	☐	☐	☐	☐	☐	☐
**37.** Apenas utilizarei teleconsultas em ambiente domiciliário se houver apoio e assistência técnica, em caso de dificuldades no uso (Kohnke et al., 2014).	☐	☐	☐	☐	☐	☐	☐
**38.** Tenciono recorrer a teleconsultas em ambiente domiciliário quando esta tecnologia e serviço estiver disponível no meu centro de saúde/hospital (Venkatesh et al., 2003).	☐	☐	☐	☐	☐	☐	☐
**39.** Tenciono recorrer a teleconsultas em ambiente domiciliário para fornecer serviços de tratamento de saúde, sempre que seja necessário (Venkatesh et al., 2003).	☐	☐	☐	☐	☐	☐	☐
**40.** **Não** pretendo recorrer de modo rotineiro a teleconsultas em ambiente domiciliário para fornecer serviços de tratamento de saúde (Venkatesh et al., 2003).	☐	☐	☐	☐	☐	☐	☐

	1	2	3	4	5	6	7
**41.** Tenciono recorrer a teleconsultas em ambiente domiciliário para fornecer cuidados de saúde, se as pessoas que influenciam o meu comportamento me incentivarem a tal.	☐	☐	☐	☐	☐	☐	☐
**42.** Tenciono recorrer a teleconsultas em ambiente domiciliário para fornecer serviços de tratamento de saúde, sempre que o doente o pretenda (Venkatesh et al., 2003).	☐	☐	☐	☐	☐	☐	☐
**43.** Tenciono recorrer a teleconsultas em ambiente domiciliário apenas se houver um incentivo financeiro inerente (Rho et al., 2014).	☐	☐	☐	☐	☐	☐	☐

Nesta secção serão apresentadas duas questões de escolha múltipla sobre teleconsultas em ambiente domiciliário. Por favor, leia os itens e responda honesta e verdadeiramente, assinalando o(s) botão(ões) da(s) opções que mais lhe parece(m) adequada(s) a cada questão. Pode assinalar mais que uma opção.

44. As teleconsultas em ambiente domiciliário correspondem a um bom método de prestação de cuidados de saúde ao ser utilizado em:

☐Primeira consulta
☐Consultas de acompanhamento/controlo
☐Consultas de emergência/não programadas
☐Nenhuma das opções anteriores
☐Outro.
Qual?____

45. Pretendo recorrer a teleconsultas em ambiente domiciliário em:

☐Primeira consulta
☐Consultas de acompanhamento/controlo
☐Consultas de emergência/não programadas
☐Não pretendo recorrer a teleconsultas em ambiente domiciliário
☐Outro.
Qual?____

------------------FIM DO QUESTIONÁRIO------------------

MUITO OBRIGADA PELA SUA COLABORAÇÃO!

Referências bibliográficas

Demiris, G., Speedie, S., & Finkelstein, S. (2000). A questionnaire for the assessment of patients' impressions of the risks and benefits of home telecare. *Journal of Telemedicine and Telecare*, *6*, 278–284. https://doi.org/10.1258/1357633001935914

Kifle, M., Payton, F. C., Mbarika, V., & Meso, P. (2010). Transfer and Adoption of Advanced Information Technology Solutions in Resource-Poor Environments: The Case of Telemedicine Systems Adoption in Ethiopia. *Telemedicine and E-Health*, *16*(3), 327–343. https://doi.org/10.1089/tmj.2009.0008

Kohnke, A., Cole, M. L., & Bush, R. (2014). Incorporating UTAUT predictors for understanding home care patients' and clinician's acceptance of healthcare telemedicine equipment. *Journal of Technology Management and Innovation*, *9*(2), 29–41. https://doi.org/10.4067/S0718-27242014000200003

Ministério da Saúde. (2013). Despacho n.o 3571/2013. Diário Da República, 2.a série(N.o 46), 8325–8326. Retrieved from https://dre.pt/dre/detalhe/despacho/3571-2013-1759945

Rho, M. J., Choi, I., & Lee, J. (2014). Predictive factors of telemedicine service acceptance and behavioral intention of physicians. *International Journal of Medical Informatics*, *83*(8), 559–571. https://doi.org/10.1016/j.ijmedinf.2014.05.005

Venkatesh, V., Morris, M. G., Davis, G. B., & Davis, F. D. (2003). User acceptance of information technology: toward a unified view. *MIS Quarterly*, *27*(3), 425–478. https://doi.org/10.2307/30036540

World Health Organization. (1998). A Health Telematics Policy. Report of the WHO Group Consultation on Health Telematics - A Health Telematics Policy. Retrieved from https://apps.who.int/iris/handle/10665/63857
