# Supplementary material for: Perceptions of Patients and Physicians on Teleconsultation at Home for Diabetes Mellitus: Survey Study
Source: JMIR Hum Factors. 2021 Nov 23;8(4):e27873. doi: 10.2196/27873 (PMC8663635; doi:10.2196/27873)
Supplement: Multimedia Appendix 2 [file humanfactors_v8i4e27873_app2.pdf]

Construct	Dimension	Observed variables/measurement items		Patients	Physicians	Wilcoxon Mann-Whitney
Expected performance	Perceived usefulness (Davis, 1989)<br>*The extent to which users believe that applying telehealth can enhance their quality of life as well as better health care service.*	P_1	Medical consultation will be completed faster	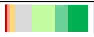	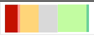	P[b]
		P_2	Improves own productivity	N/A[a]	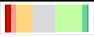	—
		P_3	Improves management of patient care	N/A	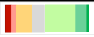	—
		P_6	Improves the effectiveness of my work	N/A	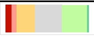	—
		P_7	Will not save me money	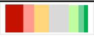	N/A	—
		P_8	Will save me time	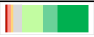	N/A	—
	Relative vantage (Li et al., 2013).<br>*Degree of perceived advantage of the individual in relation to the use of a new technology in relation to the previous practices adopted and accepted by her/him.*	P_4	Improves the patient's health	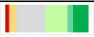	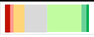	=
		P_5	Patient examination is as good as in face-to-face consultations	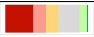	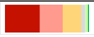	=
Expected effort	Perceived ease of use (Davis et al., 1989)<br>*The extent to which users perceive that telehealth is easy to use.*	E_1	The medical problem can be correctly understood	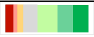	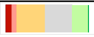	P
		E_2	I can explain my medical problems through the computer	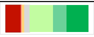	N/A	—
		E_3	Will only use teleconsultation if easy to learn	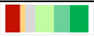	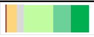	Ph[c]
		E_4	Will only use teleconsultation if easy to use	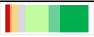	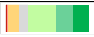	=
Social influence	Organizational support (Dansky et al., 1999).<br>*The degree of perception by a health care provider that its organizational culture encourages and supports the use of new Telemedicine technologies.*	S_1	Will only use teleconsultation if there is technical assistance	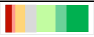	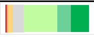	Ph

Construct	Dimension	Observed variables/measurement items		Patients	Physicians	Wilcoxon Mann-Whitney
	Coercion (Kifle et al., 2006) *The individual's perception that the people who are important to her/him think s/he should or should not adopt the new health technology.*	S_2	Will only use teleconsultation if the persons that influence my behavior encourage me	N/A	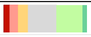	—
		S_3	Will have teleconsultation whenever the patient/physician wants to	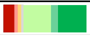	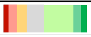	P
Facilitating conditions (Li et al., 2013)	Physician–patient relationship *The individual's perception that the use of Telemedicine technologies is perceived as disrupting the doctor–patient relationship.*	F_1	Facilitates contact with the patient/physician	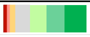	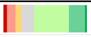	=
		F_2	Will be beneficial to manage patients and their treatment/own disease	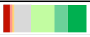	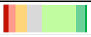	=
	Privacy of patient's data	F_3	Can invade patient's privacy	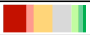	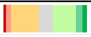	P
		F_4	Use will not interfere with confidentiality of patient's health data	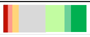	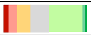	=
	Financial constraints *The individual's perception relatively to the monetary costs necessary to implement and adopt Telemedicine technology.*	F_5	Can decrease National Health System costs	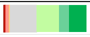	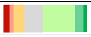	=
		F_6	Will use if there is a financial reward	N/A	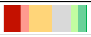	—
		F_7	Will not use if there are additional costs	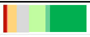	N/A	—
Attitude (Holden & Karsh, 2010)	*An individual's value judgment in relation to a certain specific behavior or action.*	A_1	It is a good way to provide health care services	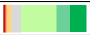	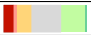	P
		A_2	It is a good idea to use teleconsultations at home	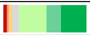	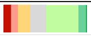	P
		A_3	It will be unpleasant in the physician–patient relationship to use teleconsultation	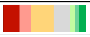	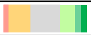	=
		A_4	In the future, teleconsultation will be a common method for providing health care services	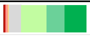	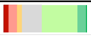	P
		A_5	Can be a supplemental health care service	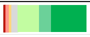	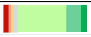	P

Construct	Dimension	Observed variables/measurement items		Patients	Physicians	Wilcoxon Mann-Whitney
		A_6	Will not increase the provision of health care services	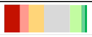	N/A	—
Intention of use (Taylor & Todd, 1995)		I_1	Will use when the required Information and Communication Technology (ICT) infrastructure is available	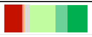	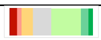	=
		I_2	Will use whenever necessary	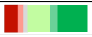	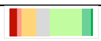	=
		I_3	Will not use routinely	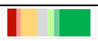	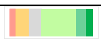	=
Demographic variables	Age	B_1		51 y (average)	36 y (average)	
	Gender	B_2		51% female	59% female	
	Qualifications	B_3		52% lower than 3rd cycle	77% general medicine	
Medical consultation	Type of patient(s)	M_1		59% type 2	(On average) 35% of the physicians' patients, over 30 patients per month, had diabetes mellitus (DM)	
	No. of DM face to face consultations	M_2		3.18 per y (Average)	(On average), 34% of all consultations, over 30 per mon	
	Time spent per consultation (average minutes)	M_3		133.5 (average, includes travel and waiting time)	23.53 (Average)	
Confidence relatively to ICT use (Lam et al., 2014)		C_1	Confidence in using a computer	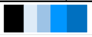	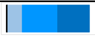	Ph
		C_2	Confidence in using internet	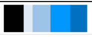	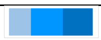	Ph
		C_3	Confidence in making videocalls	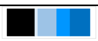	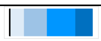	Ph

Scales: Concordance: 1, ■—Strongly disagree; 2, ■—Mostly disagree; 3, ■—Slightly disagree; 4, ■—Neither agree nor disagree; 5, ■—Slightly agree; 6, ■—Mostly agree; 7, ■—Strongly agree; Confidence: 1, ■—Not at all confident; 2, ■—Only slightly confident; 3, ■ –Moderately confident; 4, ■—Very confident; 5, ■—Extremely confident; Wilcoxon Mann-Whitney test Physicians versus Patients (group that has statistically significantly higher score): =—both groups identical.

[a] N/A: not applicable.

[b]P: patient group scored higher.

[c]Ph: physicians group scored higher.

References

Dansky, K. H., Gamm, L. D., Vasey, J. J., & Barsukiewicz, C. K. (1999). Electronic Medical Records: Are Physicians Ready? *Journal of Healthcare Management*, *44*(6), 440-454. https://journals.lww.com/jhmonline/Fulltext/1999/11000/Electronic_Medical_Records__Are_Physicians_Ready_.7.aspx

Davis, F. D. (1989). Perceived Usefulness, Perceived Ease of Use, and User Acceptance of Information Technology. *MIS Quarterly*, *13*(3), 319-340. https://doi.org/10.2307/249008

Davis, F. D., Bagozzi, R. P., & Warshaw, P. R. (1989). User Acceptance of Computer Technology: A Comparison of Two Theoretical Models. *Management Science*, *35*(8), 982-1003. www.jstor.org/stable/2632151

Holden, R. J., & Karsh, B. T. (2010). The technology acceptance model: its past and its future in health care. *J Biomed Inform*, *43*(1), 159-172. https://doi.org/10.1016/j.jbi.2009.07.002

Kifle, M., Mbarika, V. W. A., & Datta, P. (2006). Interplay of cost and adoption of tele-medicine in Sub-Saharan Africa: The case of tele-cardiology in Ethiopia. *Information Systems Frontiers*, *8*(3), 211-223. https://doi.org/10.1007/s10796-006-8780-2

Lam, M. K., Nguyen, M., Lowe, R., Nagarajan, S. V., & Lincoln, M. (2014). "I can do it": does confidence and perceived ability in learning new ICT skills predict pre-service health professionals' attitude towards engaging in e-healthcare? *Stud Health Technol Inform*, *204*, 60-66.

Li, J., Talaei-Khoei, A., Seale, H., Ray, P., & Macintyre, C. R. (2013). Health Care Provider Adoption of eHealth: Systematic Literature Review. *Interact J Med Res*, *2*(1), e7. https://doi.org/10.2196/ijmr.2468

Taylor, S., & Todd, P. A. (1995). Understanding Information Technology Usage: A Test of Competing Models. *Information Systems Research*, *6*(2), 144-176. http://www.jstor.org/stable/23011007
